# Size Matters: Small Circulating Tumor Cells Indicate Worse Therapy Response in Breast Cancer

**DOI:** 10.1002/mco2.70468

**Published:** 2025-11-11

**Authors:** Robert Wenta, Anna Muchlińska, Grażyna Suchodolska, Elżbieta Senkus, Anna Żaczek, Natalia Bednarz‐Knoll

**Affiliations:** ^1^ Laboratory of Translational Oncology, Intercollegiate Faculty of Biotechnology Medical University of Gdańsk Gdańsk Poland; ^2^ Department of Oncology and Radiotherapy Medical University of Gdańsk Gdańsk Poland

**Keywords:** cell morphology, circulating tumor cells, imaging flow cytometry, therapy response

1

Dear Editor,

The detailed phenotypic and morphological characterization of circulating tumor cells (CTCs) expands our understanding of tumor progression and may help to pinpoint clinically relevant cells for targeted intervention. Imaging flow cytometry (imFC), a cutting‐edge technology enabling standardized, high‐resolution, multiparametric CTC analysis, might significantly advance this field [1]. Its application in liquid biopsy has already not only deepened insights into epithelial‐mesenchymal transition (EMT)‐related CTCs, but also shown that CTCs vary in size and shape, including very small, largely (i.e., >80%) EMT‐like CTCs^2^. Of note, smaller tumor cell size and higher nucleus‐to‐cytoplasm ratios are also assumed as one of the frequent characteristics of stem cells, which phenotype often overlaps with EMT, and were linked to EMT phenotype in in vitro models [2, 3]. Moreover, morphological features such as irregular shape, protrusions, and micronuclei, hitherto overlooked in standard analyses but described in our recent study [2], may reflect aggressiveness, motility, and DNA damage. All of these measurable features seem to appear across multiple tumor types, albeit with varying frequency [2].

In order to test clinical relevance of those morphological characteristics (size, i.e. diameter; shape, i.e. circularity, elongation; presence of protrusions and micronucleus), in particular in the context of clinical outcome and metastasis formation, we reanalysed them in CTCs of various epithelial and EMT‐related phenotypes collected from peripheral blood of 210 breast cancer patients, and examined using multimarker immunofluorescent staining and imFC described before [2, 4] (Supporting Information).

In order to study CTC morphology independent of treatment, we excluded patients changing or undergoing therapy. We detected 408 single and 28 clustered CTCs in 52 of 187 (∼33%) patients therapy‐naïve at baseline. CTC presence correlated with older age (χ^2^ = 5.006, *p* = 0.025), showed a trend toward association with metastasis (χ^2^ = 3.452, *p* = 0.063), but no other clinical parameters. The cells displayed one of four major phenotypes, i.e., epithelial (keratin‐positive, K+V‐), epithelial‐mesenchymal (both keratin‐ and vimentin‐positive, K+V+), mesenchymal (vimentin‐positive, K‐V+), and negative for both markers (K‐V‐) (Figure 1A). K+V‐ CTCs occurred to be the most prevalent subtype and constituted the majority of large CTCs (>11.81 µm), whereas EMT‐related phenotypes predominated among small CTCs (<11.81 µm, *p* < 0.0001, Figure 1A).

**FIGURE 1:**
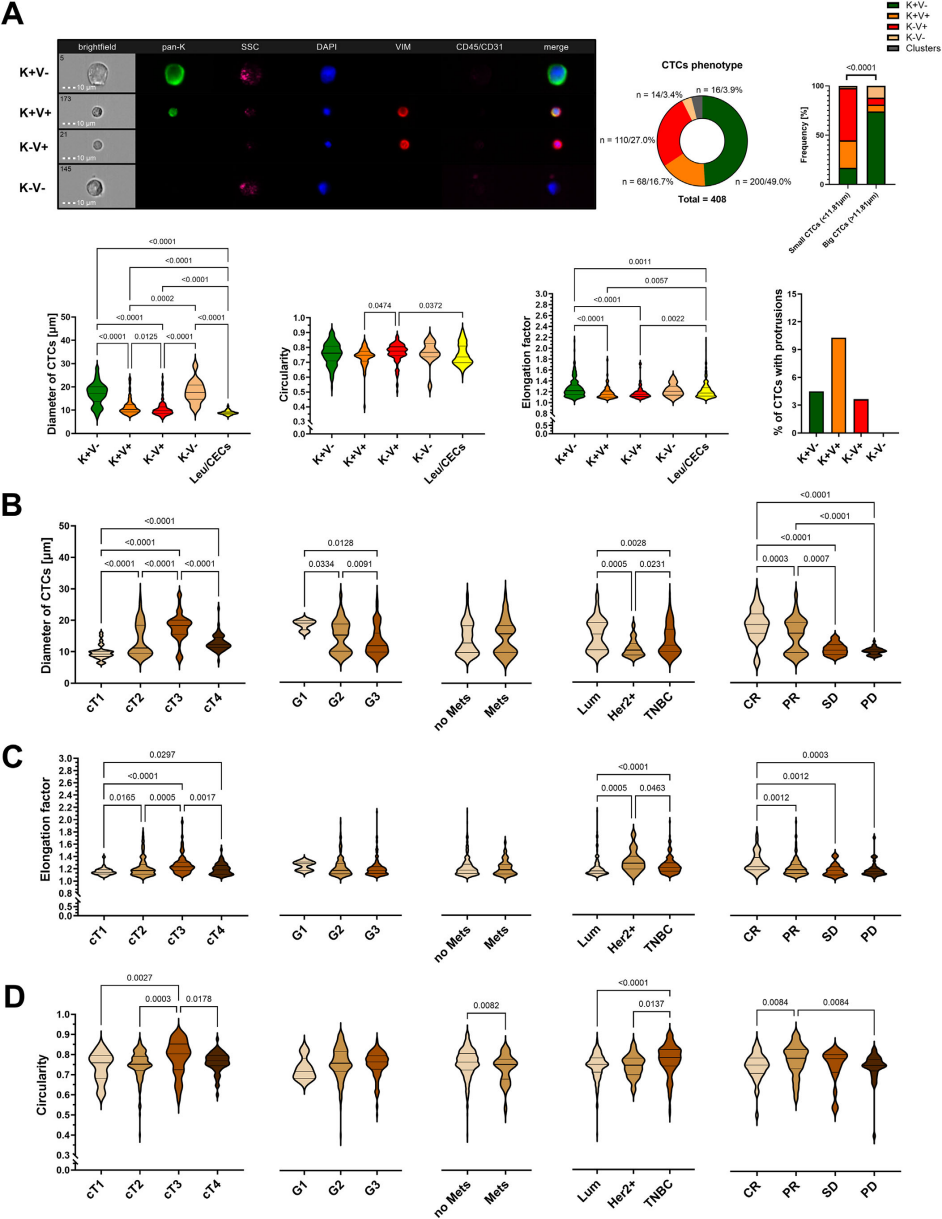
Distribution and morphometric profiling of CTC phenotypes in relation to clinical features of breast cancer. (A) Distribution of circulating tumor cells (CTCs) phenotypes in 187 breast cancer (BCa) patients (therapy‐naïve at baseline) with representative images of epithelial (K+V‐), epithelial‐mesenchymal (K+V+), mesenchymal (K‐V+), and negative for both epithelial and mesenchymal markers (K‐V‐) CTCs. Comparisons of diameters, circularity, and elongation factor between leucocytes (CD45+)/circulating endothelial (CD31+) cells (Leu/CECs, n = 400) and different CTCs (n = 408) phenotypes. The percentage of CTCs with protrusions per phenotype. Values on the graphs are presented as the number of cells/their % within the total number of cells. (B‐D) Comparative analysis of (B) diameters (size), (C) circularity and (D) elongation (shape) of identified CTCs in relation to cT, grade (G), metastasis (Mets) presence, molecular subtype (Lum ‐ uminal, Her2+ ‐ Her2‐positive and TNBC ‐ triple negative breast cancer) and treatment response (CR ‐ complete response, PR ‐ partial response, SD ‐ stable disease, PD ‐ progressive disease) of breast cancer patients. Elongation factor was calculated as the ratio of the maximum diagonal length [µm] to the minimum diagonal length, whereas circularity was the similarity of shape to a perfect circle (0–1). Statistical analysis was performed using Kruskal–Wallis or Mann–Whitney U tests, with the former followed by post hoc correction using the two‐stage linear step‐up procedure of Benjamini, Krieger, and Yekutieli. *p*‐Values less than 0.05 were considered significant. BCa indicates breast cancer, CD31 – cluster of differentiation 31, CD45 – cluster of differentiation 45, CEC ‐ circulating endothelial cell, CR ‐ complete response, cT ‐ clinical tumor stage, DAPI – 4′,6‐diamidino‐2‐phenylindole, G ‐ grade, HER2⁺ ‐ HER2‐positive breast cancer, K ‐ pan‐keratins, Leu ‐ leukocytes, Lum ‐ luminal breast cancer, Mets ‐ metastases, PD ‐ progressive disease, PR ‐ partial response, SD ‐ stable disease, SSC ‐ side scatter, TNBC ‐ triple‐negative breast cancer, V ‐ vimentin.

Overall, single CTCs were larger than leukocytes or circulating endothelial cells (Leu/CECs, Leukocytes/Circulating Endothelial Cells, cluster of differentiation 45 and/or 31+, i.e. CD45 and/or CD31+ cells, median diameter, mØ, of 12.91 µm vs. 8.82 µm, *p* < 0.0001), more circular (median circularity factor of 0.76 vs 0.74, *p* = 0.0219) and more elongated (median elongation factor of 1.28 vs 1.18, *p*<0.001, Figure 1A). K‐V‐ (mØ = 17.65 µm) and K+V‐ CTCs were found to be the largest (mØ = 17.21 µm, *p* < 0.0001), while K‐V+ were the smallest (mØ = 9.77 µm, *p* < 0.0001) (Figure 1A). K+V‐ and K‐V‐ CTCs were also most elongated (median elongation factor of 1.22/1.21, respectively, *p* < 0.0001) (Figure 1A), whereas circularity differed slightly between K‐V+ and K+V+ phenotypes (median circularity factor of 0.77/0.75, respectively, *p* = 0.0202) but was otherwise similar across phenotypes (Figure 1A). Nuclear and overall cell size were strongly correlated (Pearson correlation, *R* ^2^ = 0.875, *p* < 0.0001). Nucleus‑to‑cytoplasm ratio was highest in K+V+ CTCs (median of 0.78, *p* = 0.0023). Protrusions appeared in a small subset of CTCs across phenotype groups (4.51% / K+V‐, 10.32% / K+V+, 3.61% / K‐V+, and 0% / K‐V‐, Figure 1A), whereas micronuclei were observed in just 1.5% of CTCs and only in K+V‐ CTCs. Neither protrusions nor micronuclei correlated with clinical parameters.

Diameter (range 16.22–59.24 µm) and shape of CTC clusters were not associated with any of the analyzed clinical characteristics nor CTC phenotypes. The presence of micronuclei or cytoplasmic protrusions in the CTCs within clusters was not observed, which may be, however, biased by the difficulties in their analysis due to cluster stereometry.

Clinical tumor size (cT) correlated with CTC size and shape: larger tumors (cT3) yielded CTCs with larger diameter, higher elongation, and circularity factors compared to cT1 tumors (all p<0.001, Figure 1B–D). No significant differences in CTC morphology were observed depending on the lymph nodes' involvement (cN). Tumor grade correlated inversely with CTC size: higher grade tumors yielded the smallest CTCs (*p* = 0.0046, Figure 1B), though shape parameters did not vary significantly by grade. Metastatic tumors had more irregular CTCs defined by the lower circularity factor (*p* = 0.0082, Figure 1D).

Importantly, CTC size and shape ‐ but not its presence ‐ were influenced by molecular subtype and predictive of treatment response. Luminal subtype patients had larger (*p* < 0.0001, Figure 1B) but less elongated CTCs (*p* < 0.0001, Figure 1C), while triple‑negative breast cancer patients exhibited the most circular CTCs (*p* = 0.0001, Figure 1D). Patients achieving partial or complete response had larger (*p* < 0.0001, Figure 1B) and more elongated CTCs (*p* = 0.0003, Figure 1C), whereas those with stable or progressive disease had smaller (Figure 1B), less elongated (Figure 1C), and less circular CTCs (Figure 1D).

These findings are the first to demonstrate putative associations between pre‐treatment assessed morphology of epithelial and EMT‑related single CTCs and clinical parameters, including later response to therapy, in breast cancer. Samples were collected before therapy to avoid treatment‑induced artifacts or effects, and only cells without obvious apoptotic features were included to ensure intact morphology. The proportion of CTC‑positive patients (ca. 31%) is consistent with literature data [5]. Confirming our previous observations, single CTCs were generally larger than leukocytes, and their size correlated with primary tumor size [2]. Smaller CTCs were more common in smaller tumors and previously shown to be more frequent among EMT‐like CTCs [4].

We present the first description of morphological diversity among CTCs across breast cancer molecular subtypes, identifying luminal tumors as seeding larger and more elongated cells. The most significant observation is that larger and more elongated CTCs are associated with better response to systemic treatment, whereas smaller, largely EMT‑like, CTCs predict stable or progressive disease. This suggests that small EMT‑related CTCs may resist therapy. It might be hypothesized that at least a subpopulation of those cells might overlap with cancer stem‑like cells, which are typically small and therapy‑resistant [3].

Despite the challenges posed by low yield and high heterogeneity of CTCs, imFC ‐ a high‑throughput and multiparametric imaging platform allowing high‐resolution molecular imaging ‐ enables the detection of subtle morphological features with potential prognostic value. In conclusion, the size and morphology of CTCs correlate with clinical characteristics in breast cancer, including treatment response. Our study shows that, in particular, small CTCs are more frequently EMT‐like and potentially treatment‑resistant. Although underlying mechanisms require further investigation, these findings support the prognostic utility of quantifiable morphologic parameters such as size and shape, which can be readily assessed using standardized imaging cytometry.

## Author Contributions

Study conceptualization, design, and supervision: N.B.‐K.; patient recruitment and blood collection: E.S. and G.S.; experiments and analysis: R.W. and A.M.; data curation: R.W. and A.M.; statistics: R.W. and N.B.‐K.; resources, funding, and administrative management: A.Ż. and N.B.‐K.; draft writing: R.W. and N.B.‐K. All authors have read and approved the final manuscript.

## Funding

This research was funded by grants from the National Center for Research and Development (#WPC/33/HESCAP/2018 for Anna Żaczek) and the Ministry of Science and Higher Education, Poland (#NdS‐II/SP/0398/2023/01 for Natalia Bednarz‐Knoll).

## Ethics Statement

The study was conducted in accordance with the Declaration of Helsinki and approved by the Independent Bioethics Committee for Scientific Research at Medical University of Gdańsk (protocol no. NKBBN/748/2019‐2020, date of approval: February 5 2020).

## Consent

Written informed consent was obtained from all subjects involved in the study.

## Conflicts of Interest

2

The authors declare no conflicts of interest.

## Supporting information




**Supporting Information file 1**: mco270468‐sup‐0001‐SuppMat.docx

## Data Availability

The datasets used and/or analyzed during the current study are available from the corresponding author on reasonable request. The representative images of CTCs exhibiting various phenotypes and morphological features are deposited in the CTC Atlas (www.CTCAtlas.org).
