# Correction to “Size Matters: Small Circulating Tumor Cells Indicate Worse Therapy Response in Breast Cancer”

**DOI:** 10.1002/mco2.71109

**Published:** 2026-11-04

R. Wenta, A. Muchlińska, G. Suchodolska, E. Senkus, A. Żaczek, and N. Bednarz‐Knoll. Size Matters: Small Circulating Tumor Cells Indicate Worse Therapy Response in Breast Cancer. *MedComm* 6, no. 11 (2025): e70468. https://doi.org/10.1002/mco2.70468.

We would like to kindly request a correction to the “Funding” section of the published article. The current Funding statement does not fully comply with the funding acknowledgment required by the Ministry that supported the project.

We would therefore be grateful if the Funding section could be amended to the following wording:

Current Funding statement:

“This research was funded by grants from the National Center for Research and Development (#WPC/33/HESCAP/2018 for Anna Żaczek) and the Ministry of Science and Higher Education, Poland (#NdS II/SP/0398/2023/01 for Natalia Bednarz‐Knoll)”

Corrected Funding statement:

“Publication was funded from the State Budget under the program of the Minister of Education and Science entitled ‘Science for Society II’, project No. NdS‐II/SP/0398/2023/01, amount of funding: 1,000,000.00 PLN, total project value: 1,000,000.00 PLN (for Natalia Bednarz‐Knoll), and the program of the National Center for Research and Development entitled Poland—China Bilateral Programme, project No. #WPC/33/HESCAP/2018), amount of funding: 1,987,500.00 PLN, total project value: 1,987,500.00 PLN (for Anna Żaczek).”

This correction concerns only the Funding/Acknowledgment information and does not affect the scientific content, results, conclusions, or interpretation of the article.

We apologize for this error.

