# Supplementary material for: Size Matters: Small Circulating Tumor Cells Indicate Worse Therapy Response in Breast Cancer
Source: MedComm (2020). 2025 Nov 11;6(11):e70468. doi: 10.1002/mco2.70468 (PMC12606006; doi:10.1002/mco2.70468)
Supplement: Supplementary file 1 — Supporting Information file 1: mco270468‐sup‐0001‐SuppMat.docx [file MCO2-6-e70468-s001.docx]

**Size matters: small circulating tumor cells indicate worse therapy response in breast cancer**

**Running Head: Small CTCs Predict Poor Therapy Response**

Robert Wenta 1, Anna Muchlińska 1, Grażyna Suchodolska 2, Elżbieta Senkus 2, Anna Żaczek 1, Natalia Bednarz-Knoll 1*

1 Laboratory of Translational Oncology, Intercollegiate Faculty of Biotechnology, Medical University of Gdańsk, 80-210 Gdańsk, Poland

2 Department of Oncology and Radiotherapy, Medical University of Gdańsk, 80-211 Gdańsk, Poland

**Corresponding author**

Correspondence: Dr Natalia Bednarz-Knoll, Laboratory of Translational Oncology, Intercollegiate Faculty of Biotechnology, Medical University of Gdańsk, ul. Dębinki 1, 80-210 Gdańsk, e-mail: nbk@gumed.edu.pl

**Acknowledgements**

We thank all patients and healthy donors who agreed to donate blood. We thank Barbara Bollin-Matysiak and Olesia Zabolotna, for blood processing, Dr Beata Pieczyńska-Uziębło for technical assistance to stain samples, as well as Barbara Bollin-Matysiak for clinical data retrieval and/or curation.

**Key words**

circulating tumor cells, imaging flow cytometry, cell morphology, therapy response.

**Supplementary Information**

We enrolled 210 breast cancer patients treated at the Breast Unit at the University Clinical Center in Gdańsk between 2019 and 2022, along with 20 healthy female volunteers^1^. Thirteen patients were excluded due to ongoing therapy, leaving 187 evaluable subjects. Clinical and pathological parameters, as well as therapy response, were recorded^1^. Peripheral blood was collected before initiation of systemic treatment, and peripheral blood mononuclear cells (PBMCs) isolated via density gradient centrifugation, fixed, stored, and later stained with antibodies targeting keratins, vimentin, CD45, and CD31, followed by DAPI counterstaining as described^1^. Cells were analyzed using the Amnis ImageStreamX Mk II system (Cytek Biosciences). CTCs were gated and defined by the specialist as DAPI‑positive, CD45/CD31/CD29/aSMA‑negative cells and classified into epithelial (K+V-), epithelial‑mesenchymal (K+V+), mesenchymal (K-V+), or double‑negative (K-V-) phenotypes as described^1^ (**Fig.1A**). Morphological parameters were assessed using QuPath software on brightfield and DAPI images using area, length, circularity, maximal and minimal diameter characteristics^2^. Size and nuclear size were measured in microns [µm]; circularity ranged from 0 to 1 (with 1 representing a perfect circle); elongation was the ratio of longest to shortest diagonal; and nucleus‑to‑cytoplasm ratio was defined by nuclear length relative to the entire cell length^2^. Statistical analyses were performed using SPSS, with significance set at p<0.05. Mann-Whitney and Kruskal-Wallis tests were used to compare non-parametric variables between two or more than two groups, respectively. For Kruskal-Wallis tests, post hoc corrections were applied using the two-stage linear step-up procedure of Benjamini, Krieger, and Yekutieli to control the false discovery rate. Pearson’s correlation analysis was conducted to confirm the association between cell and nuclear sizes. Chi-squared test was used to evaluate the correlation between CTC yield and clinical parameters.

**References**

1. Muchlińska A, Wenta R, Ścińska W, et al. Improved Characterization of Circulating Tumor Cells and Cancer-Associated Fibroblasts in One-Tube Assay in Breast Cancer Patients Using Imaging Flow Cytometry. *Cancers (Basel)*. 2023;15(16):4169. doi:10.3390/CANCERS15164169/S1

2. Wenta R, Richert J, Muchlińska A, et al. Measurable morphological features of single circulating tumor cells in selected solid tumors—A pilot study. *Cytometry Part A*. Published online 2024. doi:10.1002/CYTO.A.24906
